# A clinical audit of changes in urine pH during the nutritional rehabilitation of adolescent and young adult patients hospitalised with a restrictive eating disorder

**DOI:** 10.1186/s40337-026-01681-z

**Published:** 2026-06-11

**Authors:** Sylvia Oi Yan Tang, Elizabeth Kumiko Parker, Christine Wearne, Gail Anderson, Linette Gomes, Simon Clarke, Michael R. Kohn

**Affiliations:** 1 Sydney School of Health Sciences, Faculty of Medicine and Health, The University of Sydney, Sydney, NSW 2006 Australia; 2 Department of Dietetics & Nutrition, Westmead Hospital, Westmead, NSW 2145 Australia; 3 Peter Beaumont Eating Disorders Service, Professor Marie Bashir Centre (PMBC), Sydney Local Health District (SLHD) Mental Health Services, 67-73 Missenden Road, Camperdown, NSW 2050 Australia; 4 Department of Medical Psychology, Westmead Hospital, Westmead, NSW 2145 Australia; 5 Department of Adolescent & Young Adult Medicine, Westmead Hospital, Westmead, NSW 2145 Australia; 6 Centre for Research into AdolescentS’ Health (CRASH), Westmead Hospital, Westmead, NSW 2145 Australia; 7 Sydney School of Medicine, Faculty of Medicine and Health, The University of Sydney, Sydney, NSW 2006 Australia; 8 Faculty of Health, Department of Paediatrics and Child Health, Western Sydney University, Campbelltown, NSW 2560 Australia; 9 Department of Paediatrics, Campbelltown Hospital, Campbelltown, NSW 2560 Australia

**Keywords:** Eating disorders, Urine pH, Alkaline urine, Nutritional rehabilitation

## Abstract

**Background:**

Alkaline urine pH has previously been reported in patients with restrictive eating disorders (EDs) over 30 years ago, however its prevalence is not routinely reported. This study carries out a preliminary investigation of the change in urine pH in malnourished adolescent and young adult patients hospitalised with restrictive EDs during an initial two weeks of higher energy nutritional rehabilitation.

**Methods:**

A retrospective review was carried out of the medical charts of malnourished adolescent and young adult patients (aged 14–21) hospitalised with restrictive EDs for ≥ 14 days during a 2-year period (January 2018–December 2019) and provided with assertive nutritional rehabilitation. Linear mixed effects models were used to estimate weekly within patient change in urine pH and identify possible associations with degree of malnutrition, medical instability, and purging status.

**Results:**

Seventy-nine patients (75 female, 4 male) with mean age 17.3 ± 1.4 years were included. On admission, patients were commenced on mean 2179 ± 476 kcal/day. No incidence was recorded of clinical refeeding syndrome during nutritional rehabilitation. The average decrease in urine pH ≥ 7 from baseline to 1 week of medical refeeding was statistically significant (56.6% vs. 29.9%, *p* = 0.001). Urine pH decreased from a mean of 7.0 ± 0.9 at baseline to a mean of 6.6 ± 0.7 after one week of refeeding, and further decreased to a mean of 6.5 ± 0.7 by the end of two weeks. Compared with purging patients, for non-purging patients there was a stronger association between medical instability at admission, low percentage median body mass index, and urine pH ≥ 7.

**Conclusion:**

The findings of this study suggest a change in urine pH from ≥ 7 to < 7 can be observed after one week of assertive nutritional rehabilitation in adolescent and young adults hospitalised with restrictive EDs. Further research is required to investigate potential mechanisms underlying elevated urine pH observed in patients with restrictive EDs, and the change that is observed during nutritional rehabilitation.

**Supplementary Information:**

The online version contains supplementary material available at 10.1186/s40337-026-01681-z.

## Abstract (plain language summary)

A small number of studies have reported that urine pH is elevated (pH ≥ 7) in patients with restrictive eating disorders (EDs), such as anorexia nervosa. This study retrospectively reviewed the medical charts of 79 consecutive adolescent and young adult patients, hospitalised with a restrictive ED. When comparing urine pH from admission to after one week of medical refeeding, the number of patients with urine pH ≥ 7 decreased significantly (56.6% vs. 29.9%, *p* = 0.001). The strongest association with having pH ≥ 7 was medical instability on admission, lower percentage median body mass index, and no history of purging behaviours.

The findings of this study suggest a change in urine pH from ≥ 7 to < 7 can be observed after one week of higher energy medical refeeding in a subgroup of adolescent and young adults hospitalised with restrictive EDs. Further research is needed to examine the underlying mechanisms that cause the elevation of urine pH observed in patients with restrictive EDs, and the change that is observed during medical refeeding.

**Supplementary Information:**

The online version contains supplementary material available at 10.1186/s40337-026-01681-z.

## Background

Eating disorders (EDs) are characterised by persistent disturbance of food related thoughts and emotions, and eating or eating-related behaviour. Typically, in a restrictive ED such as anorexia nervosa or atypical anorexia nervosa, there is dissatisfaction over one’s body image and an imbalance in thinking around food and body weight [1]. EDs are one of the most common chronic illnesses among adolescents, with onset usually occurring between fourteen and nineteen years of age [1]. Anorexia nervosa, in particular, can lead to serious medical complications and has been reported as having a high rate of mortality (5–10%) compared with other psychiatric disorders [2], although more recent research has shown a more positive outlook over the 30-year outcome of adolescent-onset anorexia nervosa [3].

The restrictive food intake, food aversion, purging (e.g. self-induced vomiting), and excessive exercise that can all be present in restrictive EDs may lead to malnutrition and starvation. Prolonged starvation can lead to a series of hormonal and metabolic changes to preserve muscle protein [4], which can place patients at risk of developing refeeding complications, including the refeeding syndrome [5]. Refeeding syndrome (RFS) has been described as the fluid and electrolyte shifts which occur following the rapid reintroduction of feeding to an individual who has experienced prolonged starvation, which gives rise to medical complications, including cardiac failure, seizures, confusion and even death [5, 6]. However, over the past decade, the conservative approach involving the slow rate of the reintroduction of nutrition [7], has been recognised as contributing to an ‘underfeeding syndrome’ characterised by poor weight gain during treatment [8]. The safety and efficacy of nutritional rehabilitation approaches using higher energy intakes early in the refeeding process has also been demonstrated [9–12], which has been reflected in alternative guidelines [13–15].

Starvation generally leads to increased renal ammonia production and excretion, which allows for greater acid elimination [16]. While urine can remain acidic, ammonia buffering helps prevent extreme lowering of urine pH [16]. However, rather than observing an acidic urine pH, an alkaline urine pH was previously reported in patients with restrictive EDs more than 30 years ago [17–19], and to the best of the authors knowledge, this feature has not received routine attention and reporting. Despite normal urine pH varying between 4.5 and 8.0 [20], malnourished patients with EDs have been reported to have an alkaline urine, defined as pH > 7, compared with healthy controls [17–19]. Diurnal variations in urine pH have been reported in healthy populations in studies investigating uric acid stone formation, with morning alkaline and evening acidic trends observed [21, 22]. Other possible causes of alterations in urine pH may include the limited intake of acidic food or the consumption of a vegetarian diet that is composed of lower mineral acids and protein [18, 19]. Compensatory behaviours such as chronic purging behaviours including self-induced vomiting, laxative or diuretic abuse can also influence urine pH [23], however, the impact of degree of malnutrition, presence of medical instability or purging behaviours on urine pH in the ED population remains unclear.

The aim of this study is to (i) report the change in urine pH of adolescent and young adults hospitalised with a restrictive ED, during the first two weeks of hospital admission for higher energy nutritional rehabilitation, (ii) identify possible associations with degree of malnutrition, medical instability, and purging status, and (iii) report the development of refeeding complications, including electrolyte derangements.

It is predicted that urine pH ≥ 7, will be observed in malnourished adolescents and young adults at admission to hospital with restrictive EDs, and will be restored to less than 7 early in the process of medical refeeding when provided with adequate energy intake to promote weight restoration and anabolism.

## Methods

This study is a two-year retrospective cross-sectional clinical audit of medical files of adolescent and young adults (aged 14–21 years old) hospitalised with restrictive EDs (January 2018-December 2019), at a large teaching hospital in metropolitan Sydney, Australia. This study was guided by and reported according to the ‘Strengthening the reporting of observational studies in epidemiology (STROBE) checklist for cross-sectional studies [24] (Supplementary File 1). This study has received ethics (HREC/14/WMEAD/18 (3915)) and site-specific (LNRSSA/14/WMEAD/41) approvals from the Western Sydney Local Health District Human Research Ethics Committee.

### Design and participants

Inclusion criteria were patients diagnosed with a restrictive ED, including anorexia nervosa, atypical anorexia nervosa, and Avoidant/Restrictive Food Intake Disorder (ARFID). Patients included in the study were hospitalised for nutritional rehabilitation for a minimum 14 days. Patients who were admitted for weight maintenance rather than weight restoration were excluded from the study, as the study focussed on patients provided with a higher energy refeeding protocol aiming to promote anabolism.

A sample size of 73 will have 80% power to detect a difference in means of 0.250, assuming a standard deviation of within patient change of 0.750, using a paired t-test with a 0.050 two-sided significance level.

Data was collected from the medical records on admission and during the first 2 weeks of admission and included age, sex, ED diagnosis, medical stability, history of purging behaviours, height, change in weight, change in nutrition prescription (kcal/day), blood chemistries (sodium, potassium, chloride, bicarbonate, urea, creatinine, magnesium and phosphate), clinical signs of RFS [5, 6], urine analysis [urine pH, specific gravity (SG), presence of ketones]. Only early morning urine samples were included in the analysis to improve reliability of results [25]. The urinalysis results on admission were excluded for analysis if patients were admitted in the afternoon. The first early morning urine sample was included in the analysis and regarded as the baseline data for analysis.

Patients were categorised as medically unstable if they were admitted with a heart rate lower than 50 bpm, cardiac arrhythmia, postural tachycardia, blood pressure below 80/50 mmHg, QTc interval greater than 450 msec, hypothermic (body temperature lower than 35.5 °C) or electrolyte derangement [13]. Percentage median BMI (%mBMI) was calculated for each patient using the 50th percentile of BMI-for-age-and-sex chart from Centre for Disease Control and Prevention [26].

### Ward program

Continuous nasogastric feeding would commence for medically unstable patients within the first 24 h, providing 1800–2400 kcal/day, with oral intake restricted to sips of water for the first 24 h. Once medically stable, nasogastric feeds would be reduced to cyclic nocturnal feeds, running over 10 h (2000–0600 h), and oral diet would be commenced (standardised meals plans 1800 kcal, 2300 kcal, 2800 kcal, 3300 kcal, 3800 kcal). Patients admitted medically stable were provided with an oral meal plan, with or without nasogastric feeds, and this was determined by the treating team. All oral intake was supervised by nursing staff who also provided meal support. An oral nutrition supplement drink was provided if a meal or mid meal was unfinished.

Patients were weighed 3 times/week, before breakfast and after voiding. Urinalysis was completed on weigh days using the urine sample collected prior to being weighed, and only a single urine sample was collected on weigh days.

Patients received a daily multivitamin and phosphate supplementation was empirical with the aim of maintaining a serum phosphate level of > 1.0 mmol/L. Medical monitoring included assessment of serum electrolytes (potassium, magnesium, and phosphate) at baseline, 4–6 h after initiation of nutrition, and at least daily during the first week of admission to detect and manage refeeding-related complications. Monitoring frequency was subsequently reduced to at least weekly thereafter.

The hospital normal reference ranges for blood chemistries for age and sex (Supplementary File 2) were adopted to determine whether the blood chemistries of the patients were deranged during the refeeding period.

### Statistical analysis

IBM SPSS Statistics version 25 (IBM Corp, Armonk, NY, USA) was used to analyse the data. Continuous variables were summarized using the mean and standard deviation. Categorical variables were summarized as frequencies (%). Patient profile plots were used to illustrate the change in the parameters of interest (%mBMI, urine pH, urine specific gravity (SG) and daily calorie intake) over the first two weeks following admission.

Linear mixed effects models were used to estimate the weekly ‘within patient’ rate of change and associated 95% confidence interval (95%CI) for each parameter and to investigate possible associations between the linear effect of time of diagnosis (4-level factor), medical status on admission (stable/unstable) and purging status (yes/no). In these models, patient identifier was used as the grouping variable and the continuous time variable, weeks post admission, was considered as a random and as a fixed effect. Diagnosis, medical status and purging status along with their interactions with weeks post admission were considered as fixed effects. Generalised linear mixed effect models with a logit link function were used to estimate the odds ratios of observing pH ≥ 7 across the six sampling time points and bar charts used to illustrate the percentage of patients with pH ≥ 7 at each time. Two-tailed tests with a significance level of 5% were used throughout.

## Results

**Fig. 1:**
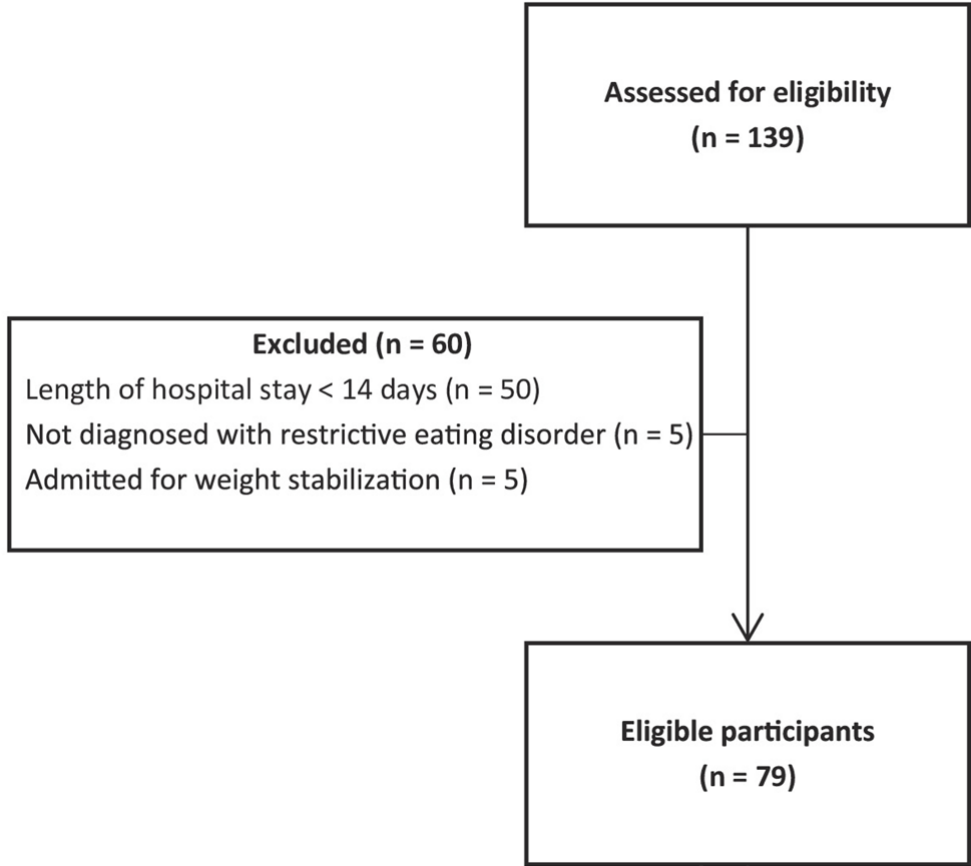
Flow diagram of patients included in this study

A total of 139 patients were admitted to the inpatient ED program in 2018 and 2019 and were assessed for eligibility for this study (Fig. 1). Among the 79 eligible participants (75 female, 4 male), the average age on admission was 17.3 ± 1.4 years, 62 (78.5%) were diagnosed with anorexia nervosa, 14 (17.7%) were diagnosed with atypical anorexia nervosa, two (2.5%) were diagnosed with ARFID, and one (1.3%) was diagnosed with an unspecified restrictive ED. Further patient characteristics and outcomes are reported in Table 1.

**Table 1:** Patient characteristics and outcomes (*n* = 79)

Variable	*N*	%
Female	75	94.9%
Age	17.3*	1.4^
Medically unstable on admission	49	62.0%
Purging behaviours	18	22.8%
Nasogastric feeding	69	87.3%
Received prophylactic phosphate supplementation	63	79.7%
Biochemistry		
Hypophosphatemia on admission	3	3.8%
Hypomagnesemia on admission	1	1.3%
Hypokalaemia on admission	3	3.8%
Developed hypophosphatemia during admission	3	3.8%
Developed hypomagnesemia during admission	5	6.3%
Developed hypokalaemia during admission	1	1.3%
Presence of urine ketones on admission	11**	15.5%
Energy intake		
Kcal/day on admission	2179*	476^
Kcal/kg/day on admission	50.3*	12.3^
Kcal/day on discharge	3565*	663^
Kcal/kg/day on discharge	69.8*	13.4^
Anthropometry		
Admission weight (kg)	44.0*	5.5^
Discharge weight (kg)	51.4*	5.6^
Average weight gain per week	1.95*	0.6^
Admission %mBMI	79.4*	7.8^
Discharge %mBMI	92.8*	6.3^
Change in %mBMI during admission	13.3*	6.2^
Length of hospital stay (days)	26.6*	9.8^

*Mean

**n = 71 for ketones analysis

^Standard deviation

One patient (1.3%) developed mild hypokalaemia (3.2 mmol/L) on day three of admission, five patients (6.3%) developed hypomagnesaemia (0.62-0.67mmol/L) during the first two weeks of admission, and three patients (3.8%) developed mild hypophosphatemia (0.68–0.72 mmol/L) during the first two weeks of admission (Table 1). In patients with deranged electrolytes, this was treated with oral replacement until levels were within the normal reference range. Three patients (3.8%) were admitted with peripheral oedema, and no patient developed peripheral oedema during the first 2 weeks of medical refeeding. All patients were discharged without peripheral oedema. No patients were diagnosed with clinical RFS using criteria defined by Rio et al. [6] or by clinical symptoms [5]. The numbers of patients with deranged levels of sodium, chloride, bicarbonate, urea and creatinine are presented in Supplementary File 3.

**Fig. 2:**
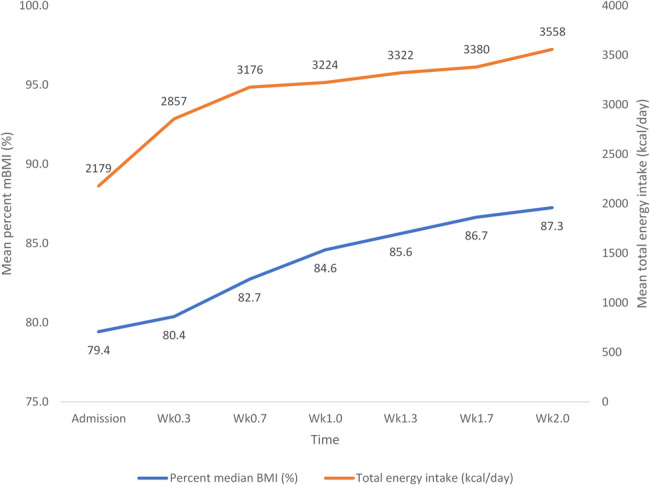
Mean total energy intake and %mBMI over initial 2 weeks of admission

The mean energy intake (kcal/day) and %mBMI increased consistently during the first two weeks of admission, indicating the calories prescribed to patients during nutritional rehabilitation were sufficient to achieve the goal of restoring weight (Fig. 2). On average, the rate of increase in %mBMI was 3.91% per week, with standard error 0.170 and *p* < 0.001.

**Fig. 3:**
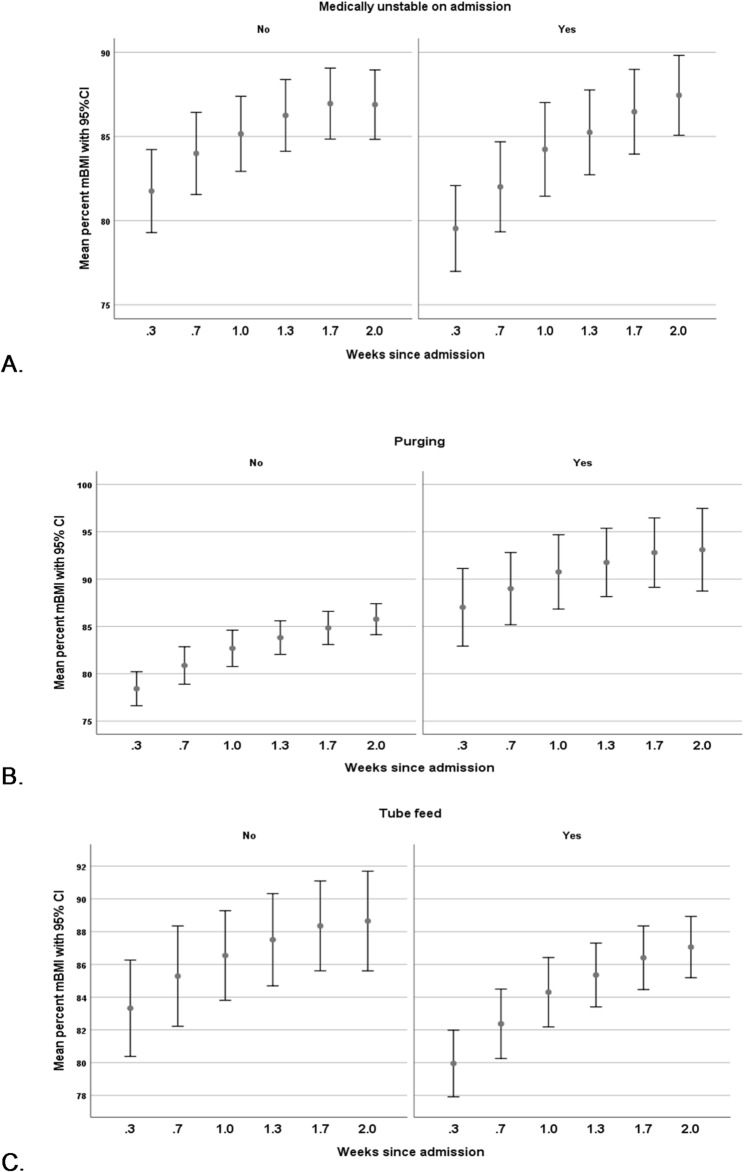
**A** Mean %mBMI and 95% CI plot over 2 weeks of admission by status of medical stability on admission. **B** Mean %mBMI and 95% CI plot over 2 weeks of admission by purging status. **C** Mean %mBMI and 95% CI plot over 2 weeks of admission by tube feeding status

The medically unstable group had a lower %mBMI on admission compared with the medically stable group. Both groups demonstrated an increase in %mBMI during the first 2 weeks of hospital admission (Fig. 3A). The mean %mBMI in the purging group was 7.74% higher than the non-purging group, indicating the non-purging group was more severely malnourished. Both the non-purging and purging groups showed an increase in %mBMI over 2 weeks of hospital admission (Fig. 3B). The non-tube feeding group had a higher mean %mBMI compared to the tube feeding group, indicating the tube fed group was more severely malnourished. Both the tube fed and the non-tube fed groups showed an increase in %mBMI over 2 weeks of hospital admission (Fig. 3C).

The number of patients with urine pH ≥ 7 reduced from 43 (56.6%) on week 0.3, which was the baseline data, to 23 (29.9%) at the beginning of week 2 (*p* = 0.001), and it further decreased to 18 (25.7%) by the end of the second week of nutritional rehabilitation (*p* < 0.001) (Table 2). There was no significant change in the odds of a patient recording a pH ≥ 7 during week 1. During week 2, the odds of a patient recording a pH ≥ 7 were approximately 66% less than at the start (*p* < 0.001).

**Table 2:** Number of patients with a urine pH result at each sampling time point and the percentage with pH ≥ 7 together with odds ratios and associated 95% CIs

Week		Number of patients with urine pH test	Number with pH ≥ 7	% patients with pH ≥ 7	Odds ratio	95%CI for OR	*p*-value
Week 1	0.3	76	43	56.6%	1	–	–
	0.7	77	38	49.4%	0.75	(0.42, 1.32)	0.318
	1.0	79	41	51.9%	0.84	(0.46, 1.54)	0.568
Week 2	1.3	77	23	29.9%	0.33	(0.16, 0.65)	0.001
	1.7	75	23	30.7%	0.34	(0.19, 0.62)	<0.001
	2.0	70	18	25.7%	0.27	(0.14, 0.51)	<0.001

The mean urine pH decreased from 7.0 ± 0.9 at baseline to 6.6 ± 0.7 after the first week of refeeding and it further decreased to 6.5 ± 0.7 by the end of the second week of nutritional rehabilitation. The mean urine pH on discharge was 6.2 ± 0.7 (Supplementary File 4).

**Fig. 4:**
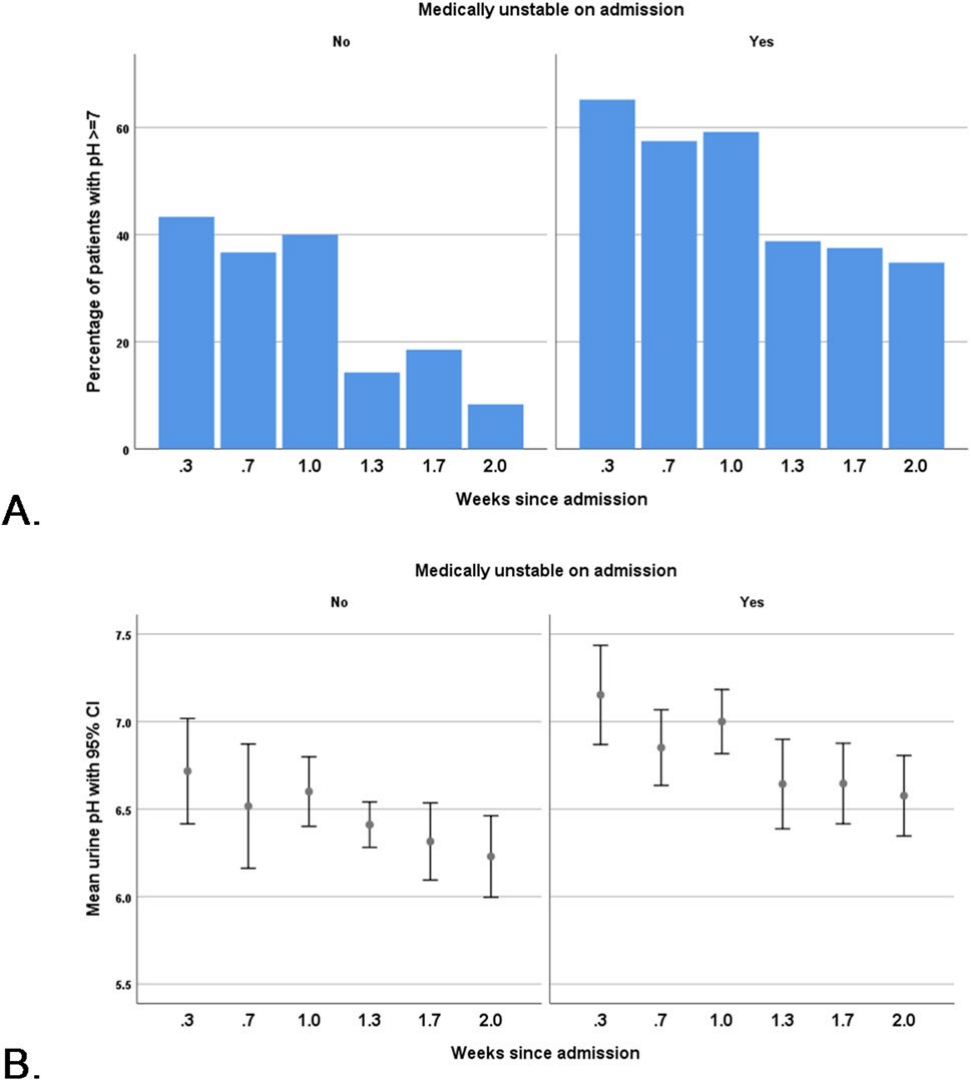
**A** Percentage of patients with pH ≥ 7 over 2 weeks of admission by status of medically stability on admission. **B** Mean urine pH and 95% CI plot over 2 weeks of admission by status of medical stability on admission

Both medically stable and unstable patients on admission displayed a significant decrease in urine pH after the first week of admission (Fig. 4A and B). However, the medically unstable group had a higher percentage of patients with urine pH ≥ 7 (65%) compared to the medically stable group (43%). Additionally, the urine pH of patients who were medically unstable at admission was 0.34 higher than those who were stable at admission. The rate of decline in urine pH was 0.32 per week for both groups, with standard error 0.06 and *p* < 0.001.

**Fig. 5:**
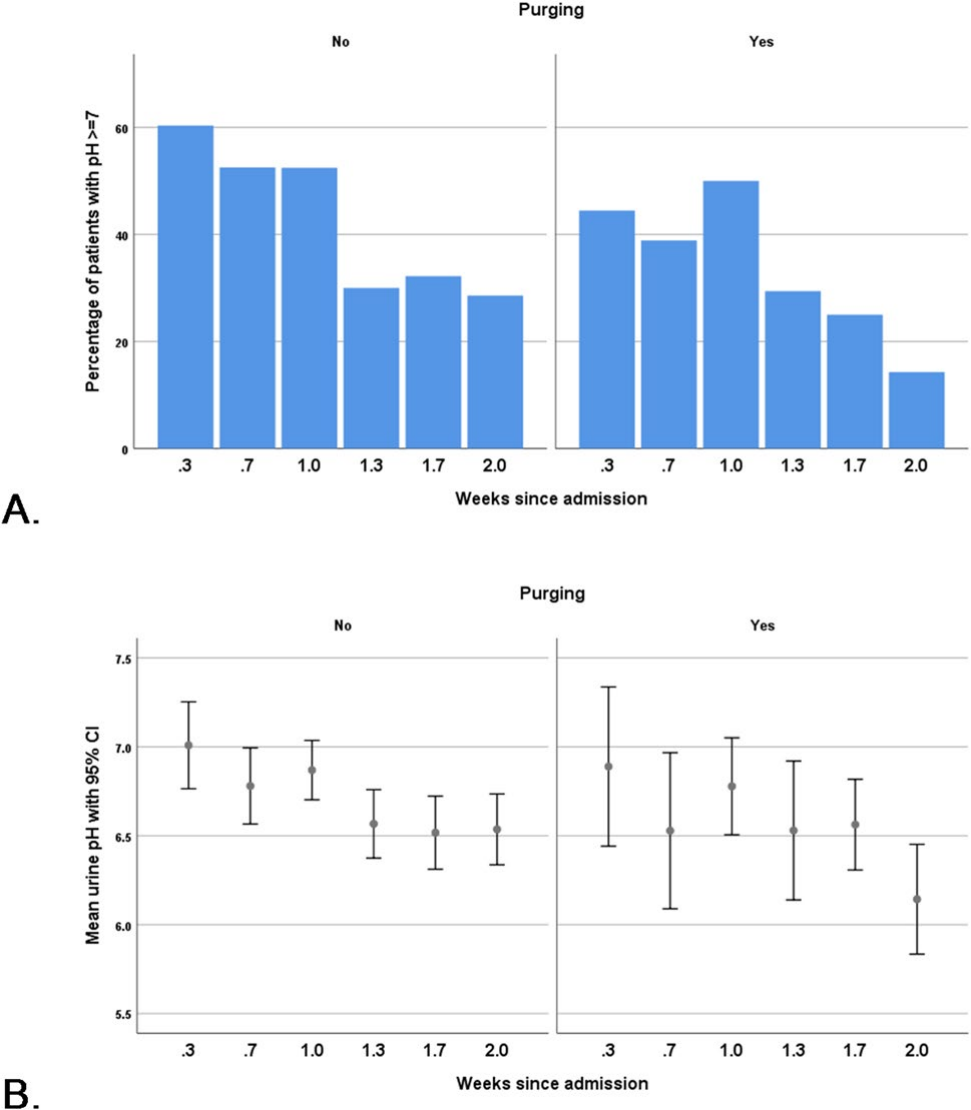
**A** Percentage of patients with pH ≥ 7 during initial 2 weeks of admission by purging status. **B** Mean urine pH and 95% CI plot over 2 weeks of admission by purging status

When comparing the change in urine pH by the purging status of patients, Fig. 5A and B show the non-purging group had a higher urine pH at baseline compared to the purging group, however a decrease in urine pH was observed in both groups after 1 week of admission.

**Fig. 6:**
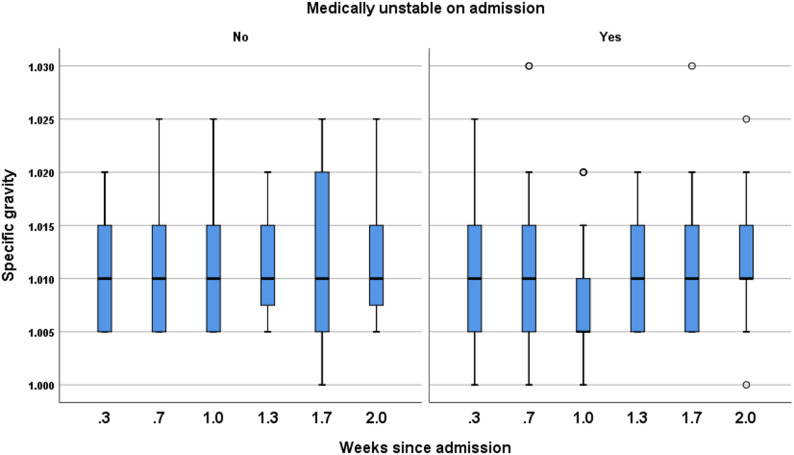
Box-and-whisker plot of change of urine SG over 2 weeks of admission by status of medical stability on admission

There was no significant association between time since admission and a patient’s urine SG status (SG ≤ 1.01 versus > 1.01, *p* = 0.132) during the first 2 weeks. Looking at the change in urine SG by medical status on admission, Fig. 6 shows the median urine SG was stable for the medically stable group and fluctuated within a tight range in the medically unstable group, hence a trend in urine SG could not be observed during two weeks of nutritional rehabilitation. Similarly, in the analysis of urine SG change by purging and tube feeding status, clear trending of urine SG could not be observed.

Although 11 out of 71 patients (15.5%) were admitted with ketones present in the urine, only 1 out of 71 patients (1.4%) had ketones detected in their urine at week 2.0. All patients were discharged without ketones present in their urine. The small number of patients with ketones present in the urine precluded any further statistical analysis on how the presence of ketones in urine could correlate with the change of urine pH observed.

The linear mixed effects model results show that %mBMI and daily calorie intake exhibited significant positive within patient linear trends over time (4.2% and 356 calories per week respectively, *p* < 0.0001). While urine pH exhibited significant negative within patient linear trend over time (-0.31 per week, *p* < 0.0001) (Table 3).

**Table 3:** Estimated within patient rate of change per week for each parameter together with 95% CI and p-value for linear trend

Parameter	Estimated slope (rate of change per week)	(95%CI for slope)	*p*-value for slope
Percent mBMI (%)	4.23	(3.89, 4.57)	< 0.0001
Urine pH	-0.31	(-0.43, -0.20)	< 0.0001
Urine specific gravity	0.00082	(-0.00005, 0.0017)	0.065
Daily energy intake (kcal)	356	(250, 461)	< 0.0001

With one exception, the linear trends over time did not depend on the factors of diagnosis, medical stability on admission or purging status (all *p* > 0.3 for interaction between linear effects of time and each factor). There was a significant interaction between the linear trend in %mBMI over time and medical stability on admission: medically stable on admission patients started their admission with higher %mBMI than medically unstable patients on admission but the weekly rate of change was less in the medically unstable group (*p* = 0.002).

The following tables set out the intercepts (estimated values at week = 0) and slopes (weekly rates of change) for each parameter by medical stability on admission (Table 4) and by purging status (Table 5).

**Table 4:** Linear mixed effects model estimates of intercepts and slopes by medical stability

Parameter	Intercept (95%CI)		Difference in intercepts *p*-value	Weekly rate of change (95%CI)		Weekly rate of change *p*-value
	Medically stable on admission	Medically unstable on admission		Medically stable on admission	Medically unstable on admission	
Percent mBMI (%)	81.2 (78.1, 84.3)	78.7 (76.3, 81.1)	0.199	3.55 (3.03, 4.08)	4.64 (4.23, 5.04)	< 0.001
Urine pH	6.84 (6.62, 7.06)	7.19 (6.99, 7.39)	0.001***	-0.32 (-0.44, -0.20) *		< 0.001
Urine specific gravity	1.011 (1.009, 1.012)	1.010 (1.008, 1.011)	0.141	0.0008 (-0.0000, 0.0017)*		0.060
Daily energy intake (kcal)	2657 (2467, 2847)	2947 (2781, 3112)	0.004**	355 (249, 460)*		< 0.001

*Common slope for medically stable and unstable on admission groups

**Estimated difference between medically unstable and stable on admission intercepts for daily calories is 290, 95%CI (98, 481)

***Estimated difference between medically unstable and stable on admission intercepts for pH is 0.35, 95%CI (0.14, 0.55)

**Table 5:** Linear mixed effects model of intercepts and slopes by purging status

Parameter	Intercept (95%CI)		Difference in intercepts *p*-value	Weekly rate of change (95%CI)		Weekly rate of change *p*-value
	No purging	Purging		No purging	Purging	
Percent mBMI (%)	77.9 (76.0, 79.8)	85.6 (82.2, 88.9)	< 0.001***	4.23 (3.89, 4.57) *		< 0.001
Urine pH	7.09 (6.89, 7.28)	6.94 (6.67, 7.21)	0.248	-0.32 (-0.44, -0.20) *		< 0.001
Urine specific gravity	1.009 (1.008, 1.011)	1.012 (1.011, 1.014)	0.001**	0.0008 (-0.0000, 0.0017)*		0.055
Daily energy intake (kcal)	2836 (2673, 2999)	2833 (2596, 3070)	0.975	356 (250, 461)*		< 0.001

*Common slope for purging and no purging groups

**Estimated difference between purging and no purging intercepts for urine specific gravity is 0.003, 95%CI (0.001, 0.005)

***Estimated difference between purging and no purging intercepts for percent mBMI is 7.7, 95%CI (4.0, 11.4)

## Discussion

This study identified that 56.6% of malnourished patients hospitalised with restrictive EDs had a urine pH ≥ 7 at the baseline of nutritional rehabilitation, and a decrease in urine pH from 7.0 ± 0.9 at baseline to 6.6 ± 0.7 was observed (*p* < 0.001), after one week of medical refeeding commencing patients on an average 2179 ± 476 kcal/day.

No patients developed clinical RFS, with only 3 patients (3.8%) developing mild hypophosphatemia which was treated with oral replacement. Our findings are consistent with the results of other studies reporting that higher energy prescription, under close medical monitoring and replacement of electrolytes when required, is effective in weight restoration without the development of RFS [9–12].

Currently, a small number of published abstracts or reports compare the difference of urine pH between patients with EDs and healthy controls on admission [17–19]. However, to the authors knowledge, this is the first study of its kind to report on the change of urine pH in patients with restrictive EDs during nutritional rehabilitation. This study has demonstrated that over 55% of malnourished patients with restrictive EDs had urine pH ≥ 7, which concur with the findings of Arden et al. who reported 37% patients with EDs admitted with urine pH > 7 while only 4% in the healthy control group had urine pH > 7 (*p* = 0.001) [18]. Although, in the current study, despite urine pH ≥ 7 decreasing to 29.9% after one week of medical refeeding, and 25.7% by the end of two weeks, the percentage of patients with urine pH ≥ 7 after two weeks of nutritional rehabilitation was still not comparable to that in the healthy controls as reported by Arden et al. [18]. This suggests that some patients may require more than two weeks of medical refeeding before their urine pH returns to pH < 7.

This study provides evidence that patients hospitalised with medical instability, who typically had a lower %mBMI, and non-purging behaviour had a stronger association with urine pH ≥ 7 at baseline than those who were medically stable and diagnosed with purging behaviours. Our findings support Robson et al.’s hypothesis that chronic purging could be one of the factors that give rise to an acidic urine pH [23].

Ardem et al. reported significantly higher urine pH in patients with EDs (*n* = 46) presenting to an adolescent medicine clinic compared with healthy controls (*n* = 28) (6.8 ± 0.8 vs. 5.9 ± 0.8, *p* < 0.001) [18]. Similarly in a study of adolescents hospitalised with anorexia nervosa or bulimia nervosa, St.Victor et al. reported urine pH was significantly higher in patients with EDs (*n* = 36) on admission compared with controls (*n* = 23) (6.6 ± 0.8 vs. 5.5 ± 0.7, *p* < 0.001) [19].

Clinicians may typically expect urine pH to be more acidic prior to nutritional rehabilitation in malnourished individuals, reflecting a catabolic state in which fat and protein breakdown generate ketone bodies and other organic acids [16]. Conversely, during nutritional rehabilitation - particularly with higher-energy refeeding protocols - the shift from fat oxidation to carbohydrate metabolism causes ketone bodies to fall and a reduction in acid production and excretion [16]. This would generally be expected to result in a rise in urine pH toward a more neutral or alkaline range. However, the current study supports previous reports that in ED populations a more alkaline urine can be observed prior to nutritional rehabilitation. This apparent contradiction reflects that other physiological factors may shift urine pH towards alkalinity and highlight the importance of interpreting urine pH within its broader clinical and behavioural context and underscore the need for further research to elucidate the underlying physiological mechanisms driving a more alkaline urine pH in ED populations.

In the current study there was no clear trending observed in the change in urine SG during two weeks of nutritional rehabilitation, which can be used an indicator of fluid overload [27]. While some ED guidelines recommend routine monitoring of urine output, specific gravity, and ketones [14, 27], the authors recommend further research to investigate if urine pH may serve as an additional biomarker for identifying the transition from a catabolic to an anabolic state in malnourished individuals with restrictive EDs. The observed shift in urine pH following one week of nutritional rehabilitation is consistent with the higher-energy refeeding protocol employed, which is designed to provide sufficient nutrition to support weight restoration within the first few days of admission. However, in some patients, changes in urine pH may take longer than two weeks to emerge; in such cases, further clinical review may be warranted to confirm adequate nutritional provision, including assessment of weight change, energy intake, and the containment of treatment-interfering behaviours (e.g. falsifying weight, concealing food, tampering with enteral feeds, purging behaviours).

A limitation of this study is the absence of analysis of macronutrient intake, especially the protein component, reported by patients prior to admission, as well as provided in meals and enteral feeding formula during nutritional rehabilitation. As reported by Breslau et al. [28], the consumption of animal protein-rich diet, in which animal protein comprises higher quantity of sulphur containing amino acids – cysteine and methionine than plant-based protein, could lead to metabolic changes that increase urinary sulphate and calcium excretion and eventually result in acidic urinary excretion [28]. The examination of the correlation of macronutrients intake, especially protein, with urine pH would therefore be valuable in future studies, as well as the impact of utilising different refeeding strategies.

Another limitation of our study is that being a retrospective study design, it highly depends on the accuracy of information recorded in the medical files by health professionals. Further research, particularly prospective studies in this population, is needed to validate these findings and to determine whether urine pH trajectories are associated with other clinical factors, such as rate of weight gain, length of hospital stay, electrolyte disturbances, and markers of kidney function (e.g. creatinine changes and acute kidney injury events). Establishing these relationships may help determine whether monitoring urine pH during nutritional rehabilitation could serve as a useful adjunct to standard clinical measures -such as weight and vital signs - in evaluating the adequacy of nutritional intake and progression toward anabolism and weight restoration.

## Conclusion

Our study reports urine pH ≥ 7 was observed in over 55% of malnourished adolescent and young adult patients hospitalised with restrictive EDs. Patients admitted with medical instability, who generally had a lower %mBMI, and without purging behaviours tended to have a stronger association with urine pH ≥ 7 at baseline. The findings of this study suggest a change in urine pH from ≥ 7 to < 7 can be observed after one week of assertive nutritional rehabilitation in adolescent and young adults hospitalised with restrictive EDs.

Further research is required to validate our findings, to examine the underlying mechanisms that cause the elevation of urine pH observed in a subgroup of patients with restrictive EDs and to prospectively evaluate the change that occurs during the medical refeeding period.

## Supplementary Information

Below is the link to the electronic supplementary material.

Supplementary Material 1.

Supplementary Material 2.

Supplementary Material 3.

Supplementary Material 4.

## Data Availability

The datasets used and/or analysed during the current study are available from the corresponding author on reasonable request.

## Abbreviations

% mBMI: Percent median body mass index
ARFID: Avoidant Restrictive Food Intake Disorder
BMI: Body mass index
CI: Confidence interval
ED: Eating disorder
SD: Standard deviation
SG: Specific gravity
RFS: Refeeding syndrome
