# Supplementary material for: A clinical audit of changes in urine pH during the nutritional rehabilitation of adolescent and young adult patients hospitalised with a restrictive eating disorder
Source: J Eat Disord. 2026 Jun 11;14:191. doi: 10.1186/s40337-026-01681-z (PMC13483597; doi:10.1186/s40337-026-01681-z)
Supplement: Supplementary file 1 [file 40337_2026_1681_MOESM2_ESM.docx]

**Supplementary File 2.**

Table S2. Normal ranges for blood chemistries

| **Variable** | **Normal ranges** | | | |
| --- | --- | --- | --- | --- |
|  | **Female < 18 years old** | **Female > 18 years old** | **Male < 18 years old** | **Male > 18 years old** |
| Potassium | 3.4-4.8 mmol/L | 3.3-4.9 mmol/L | 3.3-4.9 mmol/L | 3.3-4.9 mmol/L |
| Magnesium | 0.66-1.09 mmol/L | 0.71-1.09 mmol/L | 0.69-1.09 mmol/L | 0.69-1.09 mmol/L |
| Phosphate | 0.81-1.84 mmol/L | 0.76-1.49 mmol/L | 0.76-1.49 mmol/L | 0.76-1.64 mmol/L |
| Sodium | 134-143 mmol/L | 134-143 mmol/L | 136-144 mmol/L | 136-144 mmol/L |
| Chlorine | 98-109 mmol/L | 96-109 mmol/L | 96-109 mmol/L | 96-109 mmol/L |
| Bicarbonate | 21-31 mmol/L | 23-31 mmol/L | 23-31 mmol/L | 23-31 mmol/L |
| Urea | 3.1-6.9 mmol/L | 3.1-6.9 mmol/L | 3.1-7.4 mmol/L | 3.6-7.9 mmol/L |
| Creatinine | 39-81 umol/L | 46-89 umol/L | 61-109 umol/L | 51-99 umol/L |
