# Supplementary material for: A clinical audit of changes in urine pH during the nutritional rehabilitation of adolescent and young adult patients hospitalised with a restrictive eating disorder
Source: J Eat Disord. 2026 Jun 11;14:191. doi: 10.1186/s40337-026-01681-z (PMC13483597; doi:10.1186/s40337-026-01681-z)
Supplement: Supplementary file 2 [file 40337_2026_1681_MOESM3_ESM.docx]

**Supplementary File 3.**

Table S3a and S3b show the numbers of patients with deranged levels of sodium, chloride, bicarbonate, urea and creatinine.

Table S3a. Number of patients with low levels of Na, Cl, Bicarbonate, Urea and Creatinine

| **Variable** | **Admission** | **Developed during first 2 weeks of admission** | **Discharge** |
| --- | --- | --- | --- |
| Sodium | 5 (6.3%) | 1 (1.3%) | 0 (0%) |
| Chlorine | 4 (5.1%) | 0 (0%) | 0 (0%) |
| Bicarbonate | 7 (8.9%) | 7 (8.9%) | 1 (1.3%) |
| Urea | 23 (29.1%) | 17 (21.5%) | 0 (0%) |
| Creatinine | 7 (8.9%) | 25 (31.6%) | 6 (7.6%) |

Table S3b. Number of patients with high levels of Na, Cl, Bicarbonate, Urea and Creatinine

| **Variable** | **Admission** | **Developed during first 2 weeks of admission** | **Discharge** |
| --- | --- | --- | --- |
| Sodium | 6 (7.6%) | 26 (32.9%) | 2 (2.5%) |
| Chlorine | 2 (2.5%) | 6 (7.6%) | 0 (0%) |
| Bicarbonate | 2 (2.5%) | 14 (27.7%) | 1 (1.3%) |
| Urea | 8 (1.3%) | 27 (34.2%) | 9 (11.4%) |
| Creatinine | 1 (1.3%) | 1 (1.3%) | 0 (0%) |
