# Supplementary material for: A clinical audit of changes in urine pH during the nutritional rehabilitation of adolescent and young adult patients hospitalised with a restrictive eating disorder
Source: J Eat Disord. 2026 Jun 11;14:191. doi: 10.1186/s40337-026-01681-z (PMC13483597; doi:10.1186/s40337-026-01681-z)
Supplement: Supplementary file 3 [file 40337_2026_1681_MOESM4_ESM.docx]

**Supplementary File 4.**

Table S2. Mean and SD of variables by status
